# The efficacy and safety of direct oral anticoagulants compared with vitamin K antagonist in patients with hypertrophic cardiomyopathy and atrial fibrillation

**DOI:** 10.1186/s12959-023-00562-8

**Published:** 2024-01-02

**Authors:** Si-qi Lyu, Jun Zhu, Juan Wang, Shuang Wu, Han Zhang, Xing-hui Shao, Yan-min Yang

**Affiliations:** Emergency Center, National Clinical Research Center of Cardiovascular Diseases, Fuwai Hospital, National Center for Cardiovascular Diseases, Chinese Academy of Medical Sciences and Peking Union Medical College, Beijing, China

**Keywords:** Direct oral anticoagulants, Vitamin K antagonists, Hypertrophic cardiomyopathy, Atrial fibrillation, Meta-analysis

## Abstract

**Background:**

The benefit-risk profile of direct oral anticoagulants (DOAC) therapy in patients with hypertrophic cardiomyopathy (HCM) and atrial fibrillation (AF) has not been well established yet. This study aimed to evaluate the efficacy and safety of DOAC compared with vitamin K antagonists (VKA) in patients with HCM and AF.

**Methods:**

PubMed, EMBASE, the Cochrane Library, and clinicaltrials.gov were searched to identify studies comparing DOAC with VKA in patients with HCM and AF. The primary endpoint was thromboembolic events. The relative risks and standard errors were pooled by random-effect models using the generic inverse variance method.

**Results:**

Seven observational studies involving 9395 patients were included in this meta-analysis. Compared to the VKA group, the DOAC group displayed a similar risk of thromboembolic events [RR (95%CI): 0.93 (0.73–1.20), *p* = 0.59] and ischemic stroke [RR (95%CI): 0.65 (0.33–1.28), *p* = 0.22]. The incidence of major bleeding was comparable between the two groups [RR (95%CI): 0.75 (0.49–1.15), *p* = 0.19]. Meanwhile, DOAC therapy was superior to VKA therapy in reducing the incidences of all-cause death [RR (95%CI): 0.44 (0.35–0.55), *p* < 0.001], cardiovascular death [RR (95%CI): 0.41 (0.22–0.75), *p* = 0.004], and intracranial hemorrhage [RR (95%CI): 0.42 (0.24–0.74), *p* = 0.003].

**Conclusion:**

In patients with HCM and AF, DOAC therapy was similar to VKA therapy in reducing the risk of thromboembolic events, without increasing bleeding risk. In addition, the DOAC group displayed significant advantages in reducing mortality and intracranial hemorrhage compared with the VKA group. Further randomized controlled trials are needed to provide more evidence for DOAC therapy in this population.

**Supplementary Information:**

The online version contains supplementary material available at 10.1186/s12959-023-00562-8.

## Introduction

Hypertrophic cardiomyopathy (HCM) is one of the most common hereditary cardiovascular diseases [1–4] characterized by asymmetrical myocardial hypertrophy, cardiomyocyte disarray, and interstitial fibrosis [5]. These pathophysiologic abnormalities lead to increased risks of outflow tract obstruction, heart failure, arrhythmia, stroke, and death [6]. Compared with the general population, patients with HCM are at a significantly higher risk of developing AF, which might be attributed to atrial cardiomyopathy and atrial enlargement due to left ventricular diastolic dysfunction [7–10]. Atrial fibrillation (AF) is the most common supraventricular arrhythmia in patients with HCM [1, 11].

The coexistence of HCM and AF is associated with an elevated incidence of thromboembolic events, resulting in adverse clinical outcomes and heavy healthcare burdens [7, 9, 12–14]. Current clinical guidelines recommend that patients with HCM and AF should be anticoagulated with vitamin K antagonists (VKA) regardless of their CHA_2_DS_2_-VASc scores [3, 4]. Direct oral anticoagulants (DOAC) have been recommended for patients with non-valvular AF according to evidence of their non-inferiority or superiority over VKA [5]. However, the benefit-risk profile of DOAC therapy in patients with HCM and AF has not been well established yet. Due to the lack of randomized controlled trials, high-quality evidence on the use of DOAC for primary and secondary stroke prevention in this population is still quite limited. In recent years, several observational studies regarding anticoagulant therapy in patients with HCM and AF have been published [15–21], which might shed some light on this issue. Therefore, we undertook a meta-analysis of all available studies to evaluate the efficacy and safety of DOAC compared with VKA in patients with HCM and AF.

## Methods

### Search strategies

PubMed, EMBASE, the Cochrane Library, and clinicaltrials.gov were comprehensively searched by two independent authors (LSQ and YYM) to identify studies comparing DOAC with VKA in patients with HCM and AF published before Mar 22, 2023. The main search terms included (atrial fibrillation OR atrial flutter) AND (hypertrophic cardiomyopathy OR hypertrophic obstructive cardiomyopathy OR hypertrophic nonobstructive cardiomyopathy) AND (non-vitamin K antagonist oral anticoagulant* OR non-vitamin K antagonist anticoagulant* OR direct oral anticoagulant* OR novel oral anticoagulant* OR new oral anticoagulant* OR “oral thrombin inhibitor* OR “factor Xa Inhibitor* OR DOAC* OR NOAC* OR dabigatran OR rivaroxaban OR apixaban OR edoxaban) AND (vitamin K antagonist* OR VKA OR warfarin OR coumadin OR acenocoumarol OR phenprocoumon). Moreover, the references of retrieved studies were manually searched for additional eligible studies.

### Eligibility criteria

Studies were eligible if they met the following inclusion criteria: (1) Population: patients with HCM and AF. (2) Interventions: patients received DOAC therapy versus VKA therapy. (3) Outcomes: clinical outcomes such as thromboembolic events, all-cause death, ischemic stroke, major bleeding, major or clinically relevant bleeding, gastrointestinal bleeding, and intracranial hemorrhage were reported. (4) Strategy: retrospective or prospective studies. Exclusion criteria included: (1) studies with no relevant data; (2) ongoing studies.

### Data extraction and quality assessment

Relevant data were extracted from each eligible study by two investigators (LSQ and YYM): (1) publication information: author’s names, publication year, study design; (2) study population: sample size, baseline characteristics, diagnosis; (3) intervention: therapy, dose, duration; (4) outcomes: follow-up time, incidences of the efficacy and safety endpoints. Two reviewers (LSQ and YYM) independently evaluated the quality of included studies according to the Newcastle–Ottawa score based on the assessment of selection, comparability, and outcome [22]. A Newcastle–Ottawa score of < 6 was considered as low-quality. Any discrepancy was resolved by consensus or discussion with a third reviewer (ZJ).

## Study endpoints

The primary efficacy endpoint was thromboembolic events (including stroke and systemic embolism). The secondary endpoints were all-cause death and ischemic stroke. The safety outcomes included major bleeding, major or clinically relevant bleeding, gastrointestinal bleeding, and intracranial hemorrhage defined as per each study.

### Statistical analysis

This meta-analysis was undertaken in accordance with the Preferred Reporting Items for Systematic Reviews and Meta-Analyses (PRISMA) Statement [23]. For each study, the natural logarithm of the relative risks (RR) and its corresponding standard error (SE) were calculated. RR and SE were pooled by random-effect models using the generic inverse variance method.

The Cochrane Q test and I^2^ statistic were utilized to assess heterogeneity. *P* < 0.05 for the Cochrane Q test was defined as significant, while I^2^ statistics 25–50%, 50–75%, and 75–100% were regarded as low, moderate, and high heterogeneity. Sensitivity analysis was performed by sequentially excluding each individual study and recalculating the combined estimate on the remaining studies. It helps to assess the influence of each study on the pooled risk estimate and evaluate the stability of the results. Subgroup analyses were conducted according to sample size, follow-up time, study population, and analysis model. Begg’s funnel plot and Egger’s linear regression test were utilized to evaluate publication bias. A *p*-value of < 0.05 (two-tailed) was defined as statistically significant. Review Manager, version 5.3 Windows (The Nordic Cochrane Center, The Cochrane Collaboration, 2014, Copenhagen, Denmark) and Stata 12.0 (StataCorp LP, College Station, Texas) were used for statistical analysis.

## Results

**Fig. 1:**
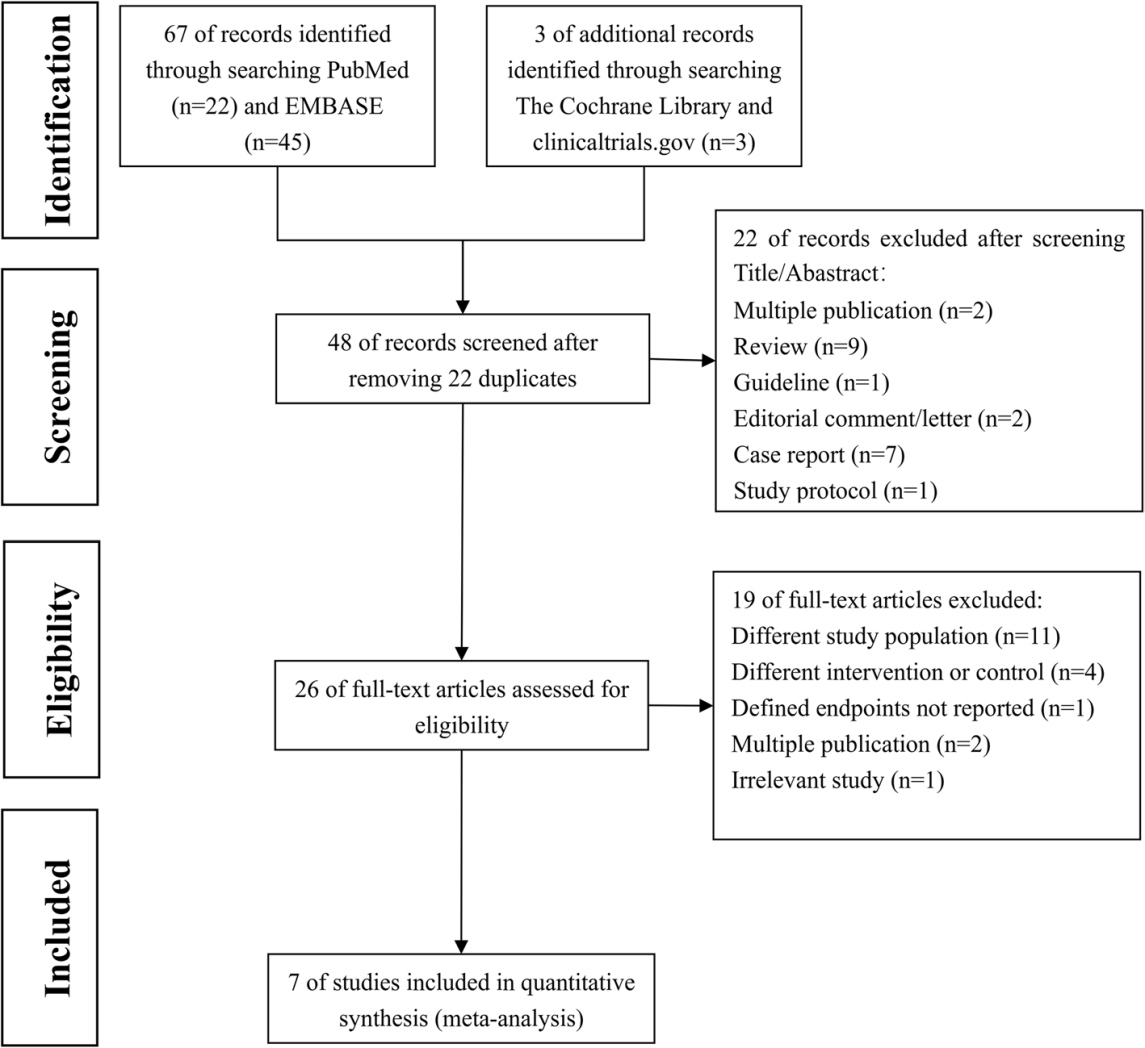
Flow diagram of the study selection process

The flow diagram of the literature retrieval process is displayed in Fig. 1. A total of 70 potentially relevant studies were initially identified. Ultimately, seven studies involving 9395 patients were included in this meta-analysis [15–21].

### Characteristics and qualities of the included studies

The baseline characteristics of the seven studies are summarized in Table 1. All of these studies were observational studies. Of the included studies, 5 studies were carried out on East Asian patients, and the rest 2 studies were conducted in Western countries. Nearly half of the studies were based on the analysis of health insurance databases. Among all included patients, 4752 patients were treated with DOAC, while 4643 patients received VKA therapy. The follow-up duration of the included studies ranged from 0.56 to 5.25 years. Subtle differences existed in the definition of endpoints across different studies. As to quality assessment, these studies were of moderate-to-high quality according to the Newcastle–Ottawa score.

**Table 1:** Detailed characteristics of included studies

Study	Country	Strategy	Source of participants	Population	Anticoagulation regimen	Endpoints		Follow-up time (years)	The Newcastle–Ottawa score
						Efficacy endpoints	Safety endpoints		
Noseworthy 2016 [15]	America	Retrospective, observational study	United States commercial insurance database (OptumLabs Data Warehouse), 2010–2015	HCM and AF (*n* = 2198)	DOACs (*n* = 579) vs. Warfarin (*n* = 1619) After propensity score matching: DOACs (*n* = 568) (Dabigatran 41.4%, Rivaroxaban 40.6%, Apixaban 18%) vs. Warfarin (*n* = 859)	Stroke or systemic embolism, ischemic stroke	Major bleeding, gastrointestinal bleeding, intracranial hemorrhage	0.56	7
Dominguez 2017 [16]	Spain	Retrospective, multicentre, longitudinal cohort study	Nine Spanish Inherited Cardiac Disease Units, 2011–2016	HCM and nonvalvular AF (*n* = 532)	DOACs (*n* = 99) (rivaroxaban 47.5%, dabigatran 29.3%, apixaban 23.2%) vs. VKAs (acenocoumarol) (*n* = 433)	Thromboembolic event (cerebrovascular accident + transient ischemic attack + peripheral embolism), death	Major or clinically relevant bleeding, gastrointestinal bleeding, intracranial hemorrhage	5.25 (2.17–9.08)	6
Jung 2019 [17]	Korea	Retrospective, observational study	Korean National Health Insurance Service database, 2011–2016	HCM and AF (*n* = 3490)	DOACs (*n* = 2302) (Rivaroxaban 39%, Dabigatran 31%, Apixaban 25%, Edoxaban 5.3%) vs. Warfarin (*n* = 1188) After propensity score matching: DOACs (*n* = 1504) vs. Warfarin (*n* = 955) The CHA_2_DS_2_-VASc score: Warfarin: 4.67 ± 2.08 DOACs: 4.82 ± 1.84	All-cause mortality, composite of fatal cardiovascular events, ischemic stroke or systemic embolism	Major bleeding, gastrointestinal bleeding, intracranial hemorrhage	1.33 ± 1.33	8
Lee 2019 [18]	Korea	Retrospective, observational study	Korean Health Insurance Review and Assessment Service database, 2013–2016	HCM and nonvalvular AF (*n* = 2397)	DOACs (*n* = 1405) (Rivaroxaban 8%, Dabigatran 22%, Apixaban 27%, Edoxaban14%) vs. Warfarin (*n* = 992) (Inverse probability of treatment weighting with propensity scores) The CHA_2_DS_2_-VASc score: Warfarin: 3.8 ± 1.9 DOAC: 3.7 ± 1.7	Ischemic stroke, all-cause death, composite outcome (ischemic stroke + all-cause death + intracranial hemorrhage + hospitalization for gastrointestinal bleeding)	Major bleeding, gastrointestinal bleeding, intracranial hemorrhage	1.60 ± 1.40	8
Park 2019 [21]	Korea	Retrospective, observational study	Samsung Medical Center, Seoul, South Korea	HCM and AF (*n* = 261)	DOACs (*n* = 158) vs. VKAs (*n* = 103)	Thromboembolic event (transient ischemic attack/stroke + peripheral embolism)	Major or clinically relevant bleeding	1.93	6
Lin 2022 [19]	China	Retrospective, single-center, observational study	The First Affiliated Hospital of Fujian Medical University, China, 2015–2019	HCM and AF (*n* = 124)	DOACs (*n* = 76) (rivaroxaban 55.3%, dabigatran 44.7%) vs. Warfarin (*n* = 48) The CHA_2_DS_2_-VASc score: Warfarin: 2 (2, 5) NOAC: 3 (2, 4)	All-cause death, cardiovascular death, thromboembolic events (ischemic stroke + TIA + left atrial thrombosis + peripheral embolism)	Clinically relevant bleeding, gastrointestinal bleeding, intracranial hemorrhage	4.47 ± 0.17	7
Liu 2022 [20]	China	Prospective, multi-center, cohort study	The Chinese Atrial Fibrillation Registry Study, 2011–2018	HCM and AF (*n* = 393)	DOACs (*n* = 133) vs. Warfarin (*n* = 260)	Thromboembolism (non-fatal ischemic stroke + peripheral embolism)	Major bleeding	3.5 (2–5)	8

*Abbreviations*: *HCM* hypertrophic cardiomyopathy, *AF* atrial fibrillation, *DOACs* direct oral anticoagulants, *VKAs* vitamin K antagonists

### Efficacy endpoints

**Fig. 2:**
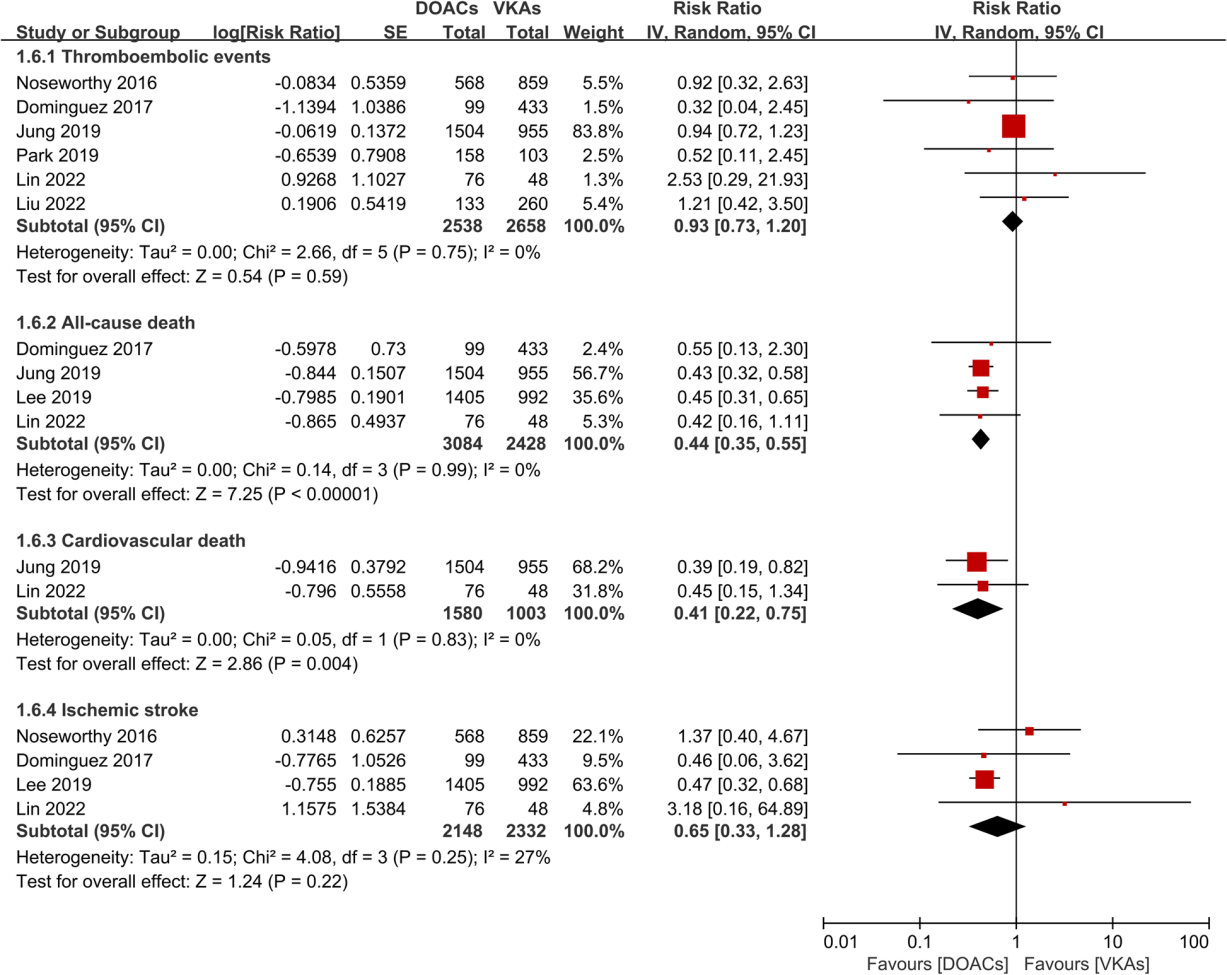
Efficacy endpoints in the DOAC group compared with the VKA group. Abbreviation: DOAC direct oral anticoagulants, VKA vitamin K antagonists

The results of the meta-analysis for the efficacy endpoints are displayed in Fig. 2. Quantitative synthesis indicated that the incidences of thromboembolic events [RR (95%CI): 0.93 (0.73–1.20), *p* = 0.59] and ischemic stroke [RR (95%CI): 0.65 (0.33–1.28), *p* = 0.22] were comparable between the DOAC group and the VKA group. But DOAC therapy was superior to VKA therapy in reducing the risk of all-cause death [RR (95%CI): 0.44 (0.35–0.55), *p* < 0.001] and cardiovascular death [RR (95%CI): 0.41 (0.22–0.75), *p* = 0.004]. Low heterogeneity existed between included studies (*P* > 0.05, I^2^ < 50%).

### Safety endpoints

**Fig. 3:**
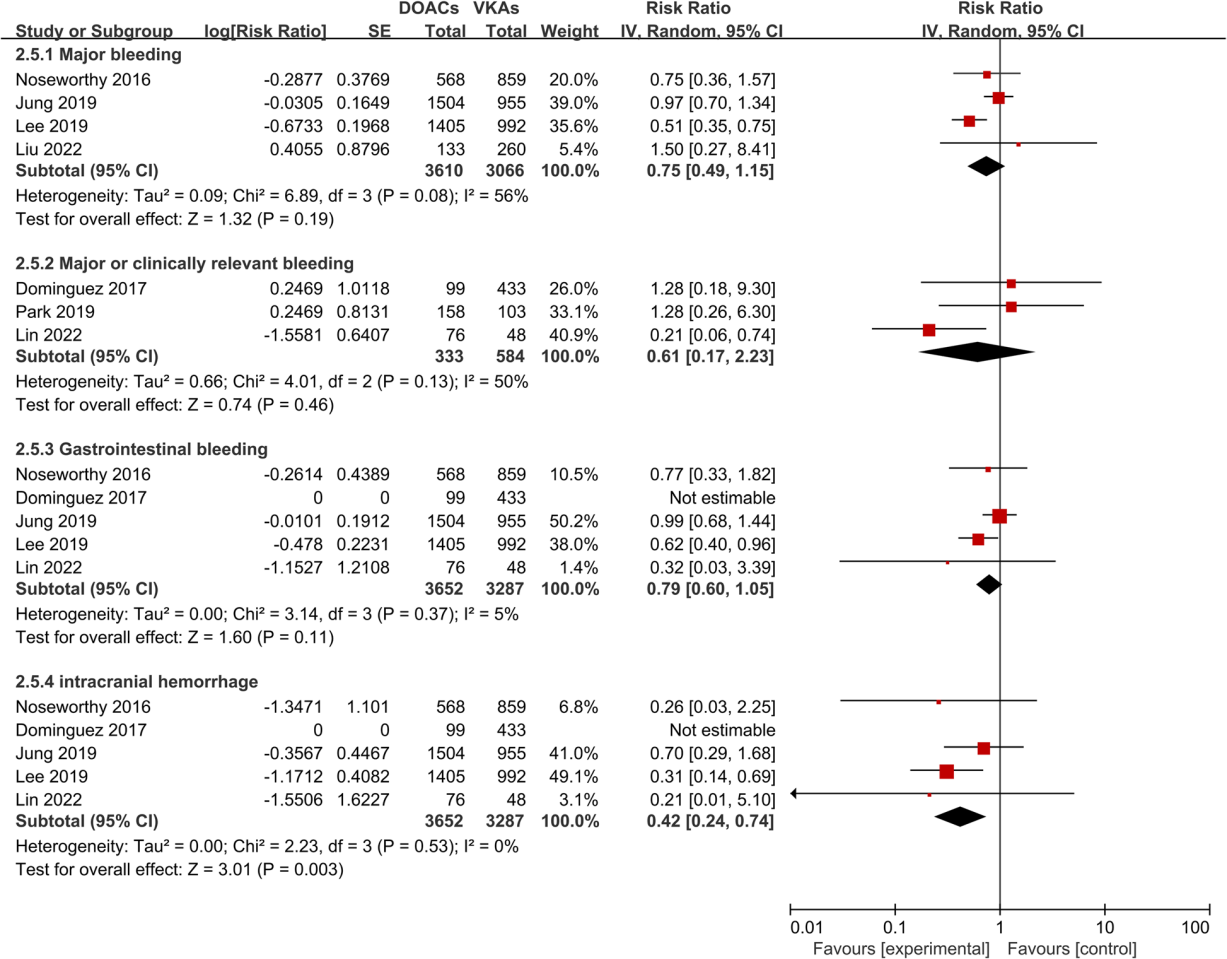
Safety endpoints in the DOAC group compared with the VKA group. Abbreviation: DOAC direct oral anticoagulants, VKA vitamin K antagonists

As to the safety endpoints (Fig. 3), there was no significant difference between the two groups in major bleeding [RR (95%CI): 0.75 (0.49–1.15), *p* = 0.19], major or clinically relevant bleeding [RR (95%CI): 0.61 (0.17–2.23), *p* = 0.46], and gastrointestinal bleeding [RR (95%CI): 0.79 (0.60–1.05), *p* = 0.11]. But DOAC therapy was related to a remarkably reduced risk of intracranial hemorrhage compared with VKA therapy [RR (95%CI): 0.42 (0.24–0.74), *p* = 0.003]. Heterogeneity between studies was low in regard to major or clinically relevant bleeding, gastrointestinal bleeding, and intracranial hemorrhage (*P* > 0.05, I^2^ ≤ 50%). But there existed moderate heterogeneity for major bleeding (*p* = 0.08, I^2^ = 56%), which might be ascribed to the diverse definitions of major bleeding in different studies.

### Sensitivity analysis and sub-group analysis

**Fig. 4:**
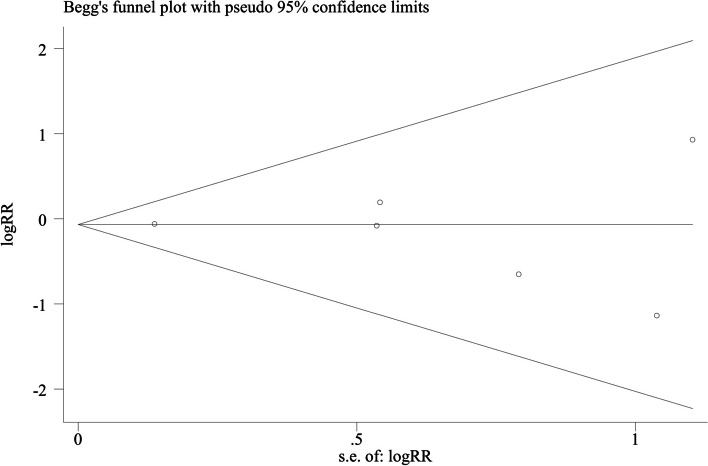
Begg’s funnel plot of publication bias in a selection of studies on the rates of thromboembolic events between DOAC and VKA therapy. Abbreviation: DOAC direct oral anticoagulants, VKA vitamin K antagonists, log (RR): natural logarithm of RR; S.E: standard error. The horizontal line means the magnitude of the effect

Sensitivity analysis demonstrated that the results of RR for thromboembolic events were stable after sequentially excluding each individual study (Shown in Supplementary Fig. 1). Subgroup analyses according to sample size, follow-up time, study population, and analysis model have come to consistent results with the overall analysis (Table 2). Begg’s Funnel plots (Fig. 4, *p* = 1.000) and Egger’s test (t = -0.28, *p* = 0.791) for the primary endpoint indicated no significant publication bias.

**Table 2:** Subgroup analysis for thromboembolic events comparing DOACs therapy with VKAs therapy

Category	Studies [references]	Patients	Pooled estimates		Test of heterogeneity	
			**RR(95% CI)**	***p* value**	**I**^2^	***p* value**
Sample size						
≥ 1000	2 [15, 17]	3886	0.94 (0.72–1.22)	0.63	0%	0.97
< 1000	4 [16, 19–21]	1310	0.90 (0.42–1.92)	0.79	0%	0.45
Follow-up time						
≥ 3 years	3 [16, 19, 20]	1049	1.07 (0.45–2.55)	0.88	0%	0.37
< 3 years	3 [15, 17, 21]	4147	0.92 (0.71–1.19)	0.54	0%	0.76
Population						
Western	2 [15, 16]	1959	0.74 (0.29–1.87)	0.52	0%	0.37
East Asian	4 [17, 19–21]	3237	0.95 (0.74–1.23)	0.70	0%	0.67
Analysis model						
Fixed effect	6 [15–17, 19–21]	5196	0.93 (0.73–1.20)	0.59	0%	0.75
Random effect	6 [15–17, 19–21]	5196	0.93 (0.73–1.20)	0.59	0%	0.75

*Abbreviations*: *DOACs* direct oral anticoagulants, *VKAs* vitamin K antagonists, *RR* odd ratio, *CI* confidence interval

## Discussion

In the present study, we have undertaken a systematic review of studies on the efficacy and safety of DOAC versus VKA in patients with HCM and AF. In this meta-analysis of 9395 patients with HCM and AF, the incidences of thromboembolic events, ischemic stroke, major bleeding, major or clinically relevant bleeding, and gastrointestinal bleeding were comparable between the DOAC group and the VKA group. In addition, DOAC therapy was superior to VKA therapy in reducing the risk of all-cause death, cardiovascular death, and intracranial hemorrhage.

AF is the most prevalent sustained arrhythmia in patients with HCM [1, 11]. During a follow-up of 10 years, about 22% to 30% of patients with HCM would develop AF [8, 9, 12]. The probability of developing AF in patients with HCM is fourfold to sixfold higher than that in the general population [7–10]. Patients with HCM have a remarkably elevated thromboembolic risk when complicated by AF. The annual incidence of stroke is estimated to be 4%, and about 27% of patients with HCM and AF would experience a thromboembolic event during their lifetime [12]. Given the severe thromboembolic risk, clinical guidelines suggested that all patients with HCM and AF should receive lifetime anticoagulation therapy when no contraindication exists. The commonly-used CHA_2_DS_2_-VASc score is not recommended for stroke risk evaluation in patients with HCM and AF [3, 4].

Previous studies showed that VKA therapy could significantly reduce the incidence of thromboembolic events in patients with HCM and AF [8, 11]. However, VKA is related to several shortcomings including narrow therapeutic windows, dosage variations, frequent monitoring, and drug-food interactions [24]. The superiority or non-inferiority of DOAC versus VKA has been confirmed in patients with nonvalvular AF [25–28]. However, evidence on the use of DOAC in patients with non-valvular AF could not be directly generalized to patients with AF and HCM since different patterns of structural cardiac abnormalities might result in variant responses to anticoagulant therapy. Previous studies demonstrated that hypertrophic obstructive cardiomyopathy might lead to blood stagnation [9, 29] and enhance the thrombogenesis of endothelium [30]. Meanwhile, it’s detected that some cell lines of HCM patients could produce thrombosis-inducing anti-cardiolipin antibodies when AF occurs [31]. These factors might contribute to the distinctive characteristics of patients with HCM and AF in thromboembolic risk and treatment response. A post-hoc analysis of the RE-LY study showed that left ventricular hypertrophy was related to reduced antithrombotic efficacy of warfarin in AF patients, but not of dabigatran [32]. The number of HCM patients included in existing DOAC trials is presumed to be low, since these patients tend to be younger and do not exhibit typical thromboembolic risk factors demanded to participate in DOAC trials [25–28]. Therefore, data on the efficacy and safety of DOAC in patients with HCM and AF is lacking. Since there is no randomized controlled trial on DOAC therapy in patients with HCM and AF, the efficacy and safety of DOAC in these patients are still controversial.

In recent years, accumulative observational studies have indicated the potential of DOAC in patients with HCM and AF, with a comparable thromboembolic risk and a reduced bleeding risk versus VKA [15–21]. Noseworthy, et al. used a large United States commercial insurance database to provide a glimpse at real-world clinical outcomes of DOAC use in patients with HCM and AF for the first time. A total of 2198 patients with HCM and AF were included. After propensity-score matching, patients treated with DOAC (*n* = 568) displayed a similar risk of stroke or systemic embolism (1.93 vs. 2.03 per 100 person-years) and a nonsignificant lower incidence of major bleeding (4.18 vs. 5.38 per 100 person-years) compared with those using warfarin (*n* = 859) [15]. A small multicenter study conducted in Spain indicated that patients receiving DOAC therapy (*n* = 99) showed similar embolic and bleeding incidences compared with those treated with VKA (*n* = 433). But patients receiving DOAC therapy reported better treatment satisfaction [16]. So far, data on this issue with the largest sample size were provided by South Korea [17, 18, 21]. Jung et al. identified 955 warfarin-treated and 1504 DOAC-treated patients with HCM and AF (1:2 propensity-matched) from the Korean National Health Insurance Service database. During a median follow-up of 16 months, the incidences of ischemic stroke and major bleeding were comparable between the two groups. But DOAC therapy was related to a remarkably lower risk of all cause-mortality [HR (95% CI): 0.43 (0.32–0.57)] and composite fatal cardiovascular events [HR (95% CI): 0.39 (0.18–0.82)] compared with warfarin therapy [17]. In another real-world Korean study involving 2397 patients with HCM and AF, DOAC was indicated to be superior to warfarin in both effectiveness and safety. This superiority was constant disregarding DOAC dose. In addition, separate analysis for individual DOAC showed that significantly reduced risks of ischemic stroke and the composite outcome could be observed in all DOAC [17]. Evidence on DOAC therapy in Chinese patients with HCM and AF was still limited. A small retrospective study including 124 Chinese patients with HCM and AF demonstrated that DOAC had a lower incidence of clinically relevant bleeding and a similar risk of all-cause death, cardiovascular death, and thromboembolic events compared with warfarin [19]. Liu et al. undertook a prospective, multicenter registry study that enrolled 393 patients with AF and HCM. During a median follow-up of 42 months, the risk of thromboembolism [(HR (95%CI): 1.21 (0.42–3.50)] and major bleeding [HR (95%CI): 1.50 (0.27–8.41)] were similar between the DOAC-treated group (*n* = 133) and the warfarin-treated group (*n* = 260) [33].

There are two meta-analyses focused on DOAC therapy in patients with HCM and AF so far [34, 35]. They were published in 2019 and 2020 respectively. Zhou et al. undertook a meta-analysis of 4 observational studies and found that DOAC therapy was associated with reduced incidences of ischemic stroke, all-cause death, and intracranial hemorrhage. But there was no difference in the risk of stroke or systemic embolism, major or clinically relevant bleeding, and gastrointestinal bleeding in DOAC-treated patients compared with VKA-treated patients [34]. Another meta-analysis of three retrospective cohort studies showed that patients receiving DOAC therapy had a significantly lower incidence of all-cause death but a similar risk of ischemic stroke, major bleeding, and intracranial bleeding compared with patients using VKA [35]. Recently, several observational studies on this issue, including two conducted on Chinese patients, have been published [19–21]. To the best of our knowledge, our meta-analysis of 7 studies has provided a comprehensive, updated, integrated conclusion on the efficacy and safety of DOAC therapy in patients with HCM and AF.

The above-mentioned evidence has contributed to the improvement of recommendation levels for DOAC in present guidelines. In the 2020 American Heart Association/American College of Cardiology Foundation guideline for HCM, anticoagulation is recommended with direct-acting oral anticoagulants (DOAC) as the first-line option and vitamin K antagonists as the second-line option in patients with HCM and clinical AF (Class I, level of evidence B) [4]. Despite the absence of randomized controlled trials, accumulative evidence from observational studies showed that DOACSs might be effective and safe in patients with HCM and AF [15–21]. Therefore, the 2021 European Heart Rhythm Association guideline recommended that patients with HCM might be eligible for DOAC therapy [36]. However, it must be pointed out that these recommendations were based on evidence from observational studies. Further randomized controlled trials on the efficacy and safety of DOAC in patients with HCM and AF might shed more light on this issue.

Several limitations should be noted in this meta-analysis. First, some of the included studies were conducted in a single center with small sample sizes and short follow-up time, leading to underpowered results and unreliable conclusions. Second, heterogeneity existed in study design and endpoint definitions across the 7 included studies, which might have an impact on the results to some extent. Third, due to the observational nature of included studies, the relevant confounders could hardly be exhaustive. The results should be interpreted with caution. In addition, due to the limited data, we could not perform the subgroup analysis according to DOAC types, DOAC dosages, time in the therapeutic range, and HCM phenotypes. Finally, although an extensive search of databases has been undertaken, some studies might not be included.

## Conclusion

In patients with HCM and AF, DOAC therapy was similar to VKA therapy in reducing the risk of thromboembolic events and ischemic stroke, without increasing bleeding risk. In addition, the DOAC group displayed remarkably decreased incidences of all-cause death, cardiovascular death, and intracranial hemorrhage compared with the VKA group. Further elaborately designed, large, multicenter randomized controlled trials are needed to provide more evidence for DOAC therapy in patients with HCM and AF.

### Supplementary Information

**Additional file 1: Supplementary Figure 1.** Sensitivity analysis for thromboembolic events by sequentially excluding each individual study.

## Data Availability

The datasets used and analyzed during the current study are available from the corresponding author on reasonable request.
