# Supplementary figures and images for: The efficacy and safety of direct oral anticoagulants compared with vitamin K antagonist in patients with hypertrophic cardiomyopathy and atrial fibrillation
Source: Thromb J. 2024 Jan 2;22:2. doi: 10.1186/s12959-023-00562-8 (PMC10759476; doi:10.1186/s12959-023-00562-8)

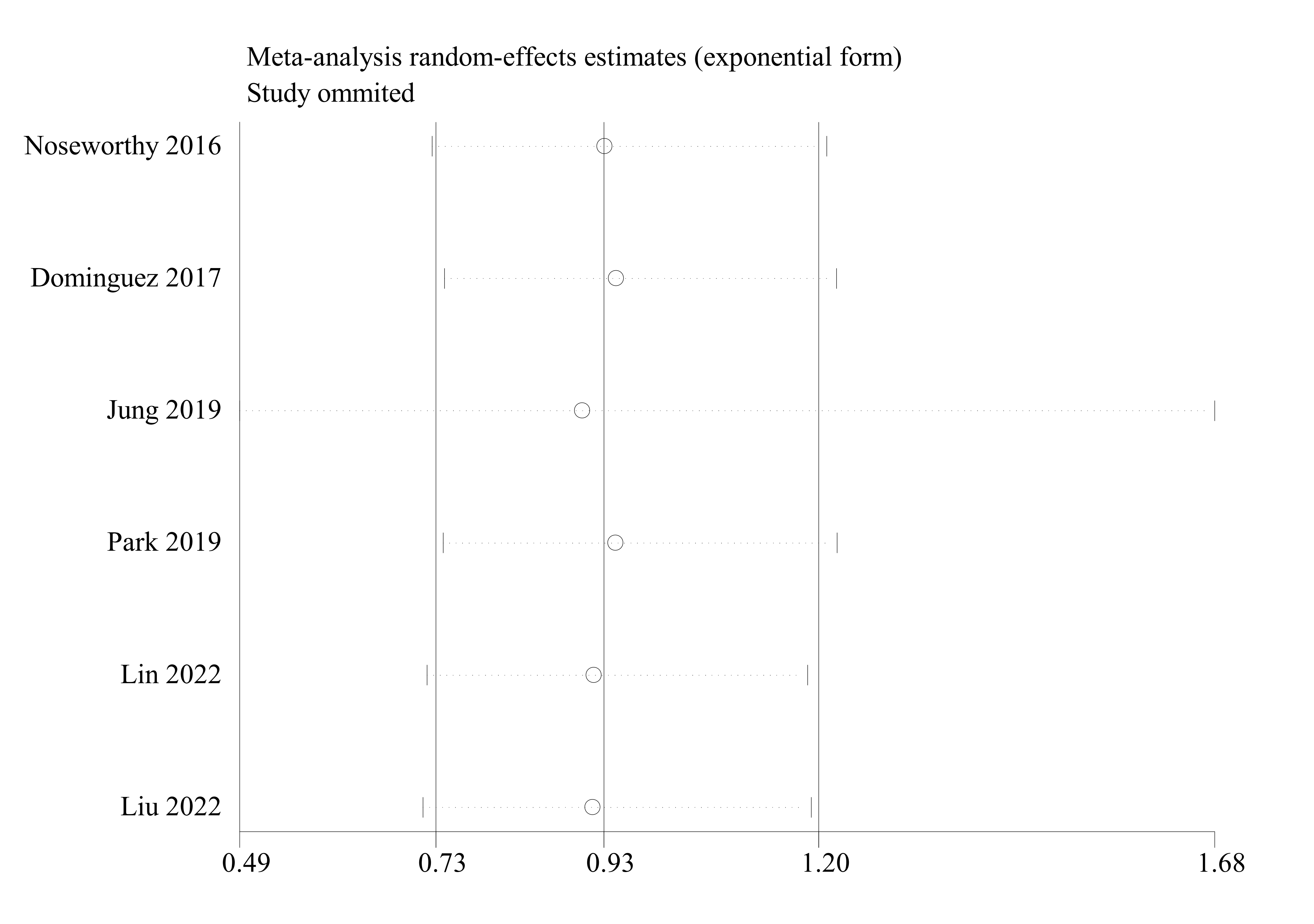

Supplement: Supplementary file 1 — Additional file 1: Supplementary Figure 1. Sensitivity analysis for thromboembolic events by sequentially excluding each individual study. [file 12959_2023_562_MOESM1_ESM.tif]
